# Pb(II) Induces Scramblase Activation and Ceramide-Domain Generation in Red Blood Cells

**DOI:** 10.1038/s41598-018-25905-8

**Published:** 2018-05-10

**Authors:** Hasna Ahyayauch, Aritz B. García-Arribas, Jesús Sot, Emilio J. González-Ramírez, Jon V. Busto, Bingen G. Monasterio, Noemi Jiménez-Rojo, F. Xabier Contreras, Adela Rendón-Ramírez, Cesar Martin, Alicia Alonso, Félix M. Goñi

**Affiliations:** 1 Instituto Biofisika (CSIC, UPV/EHU), 48080 Bilbao, Spain; 2 Departamento de Bioquímica, University of the Basque Country (UPV/EHU), 48080 Bilbao, Spain; 3 Institut Supérieur des Professions Infirmières et des Techniques de Santé, Rabat, Morocco; 4 Neuroendocrinology Unit, Laboratory of Genetics, Neuroendocrinology and Biotechnology, Faculty of Sciences, Ibn Tofail University, Kenitra, Morocco; 5 NCCR Chemical Biology, Department of Biochemistry, University of Geneva, 1211 Geneva, Switzerland

## Abstract

The mechanisms of Pb(II) toxicity have been studied in human red blood cells using confocal microscopy, immunolabeling, fluorescence-activated cell sorting and atomic force microscopy. The process follows a sequence of events, starting with calcium entry, followed by potassium release, morphological change, generation of ceramide, lipid flip-flop and finally cell lysis. Clotrimazole blocks potassium channels and the whole process is inhibited. Immunolabeling reveals the generation of ceramide-enriched domains linked to a cell morphological change, while the use of a neutral sphingomyelinase inhibitor greatly delays the process after the morphological change, and lipid flip-flop is significantly reduced. These facts point to three major checkpoints in the process: first the upstream exchange of calcium and potassium, then ceramide domain formation, and finally the downstream scramblase activation necessary for cell lysis. In addition, partial non-cytotoxic cholesterol depletion of red blood cells accelerates the process as the morphological change occurs faster. Cholesterol could have a role in modulating the properties of the ceramide-enriched domains. This work is relevant in the context of cell death, heavy metal toxicity and sphingolipid signaling.

## Introduction

Lead (Pb^2+^) is one of the most abundant heavy metals on earth. It has been widely used throughout human history, posing a serious health problem to susceptible populations. This metal causes a broad range of biochemical, physiological and behavioural dysfunctions. Pb^2+^ exposure may affect the central and peripheral nervous system, the hematopoietic and cardiovascular systems, kidney, liver and the reproductive system via physiological, biochemical and behavioural changes^1,2^. In human blood about 95% lead is accumulated in red blood cells (RBC, erythrocytes)^1^ suggesting that they could be an important target of lead toxicity in the cardiovascular system^3^. In erythrocytes lead could suppress hemoglobin synthesis^4^, induce oxidative stress^5^, inhibit superoxide dismutase, and lower intracellular glutathione^6^. Lead is also known to induce shape change in erythrocytes from normal biconcave erythrocyte to spiked echinocyte and even achantocyte^7^.

Lead has been associated with the induction of apoptosis^8^. In studies performed on lead-exposed mammal erythrocytes it was found that apoptosis presumably contributes to a decrease in the life-span of erythrocytes and the development of anaemia in cases of lead poisoning^7,9,10^. Some experiments have disclosed that injured erythrocytes expose phosphatidylserine (PS) at their surface^11^. Because macrophages are equipped with receptors specific for phosphatidylserine^12,13^, erythrocytes exposing phosphatidylserine (PS) at their surface will be rapidly recognized, engulfed, and degraded^14^ thus rapidly eliminated from circulating blood.

It has been reported that micromolar or even submicromolar Pb^2+^ concentrations cause lipid scrambling and exposure of PS to the outer membrane leaflet in erythrocytes via a scramblase action, as well as calcium entry in proteoliposomes^15^. However, the cellular mechanism of the process is not yet fully understood and previous reports from our laboratory show that the biophysical properties of RBC membranes are also altered by the effect of lead^16^. A role of flip-flop inducing lipids, such as long-chain ceramides^17,18^, has also been considered for the PS exposure mechanism. Ceramides are of particular interest for their bioactive pro-apoptotic signalling role^19,20^ and their marked effects on membrane biophysical properties with a tendency to form highly-packed segregated domains^21^, but their role in eryptosis has not been fully characterized yet^9,22,23^. However, ceramide-enriched domains have been already reported for erythrocytes under a hot-cold hemolytic process^24^. The fact that erythrocyte membranes have a large amount of cholesterol, circa 45 mol%^25^, should also be taken into consideration, as it could interfere with ceramide-enriched domains^26^. The generation of non-purely ceramidic gel domains under saturating conditions is also possible and gel phases containing both ceramide and cholesterol can appear if both lipids are saturating the membrane^27,28^ which could be the case for RBC membranes under Pb^2+^ stress. A recent study from our laboratory demonstrated that RBC lipid extracts in the presence of high concentrations of ceramide exhibit lamellar gel domains enriched in both cholesterol and ceramide^29^.

In this work we provide insights about the lead-induced eryptotic mechanism. A noticeable rise in intracellular calcium via scramblase is initially detected (linked to K^+^ depletion^30^, as tested with clotrimazole inhibitor^7,31^), followed by the generation of ceramide-enriched domains in the RBC membrane and a morphological change to achantocytes over time. This is followed by PS exposure in the outer membrane leaflet. In the next stage spherocytes are formed. Finally, when a critical amount of ceramide is reached, cell lysis occurs. Upon partial cholesterol depletion, the morphological change process became accelerated, suggesting a direct relationship between cholesterol/ceramide-enriched domains in the membrane and the change in morphology, which in turn could govern the kinetics of the eryptotic process.

## Results

### Lipid scrambling in living cells

**Figure 1:**
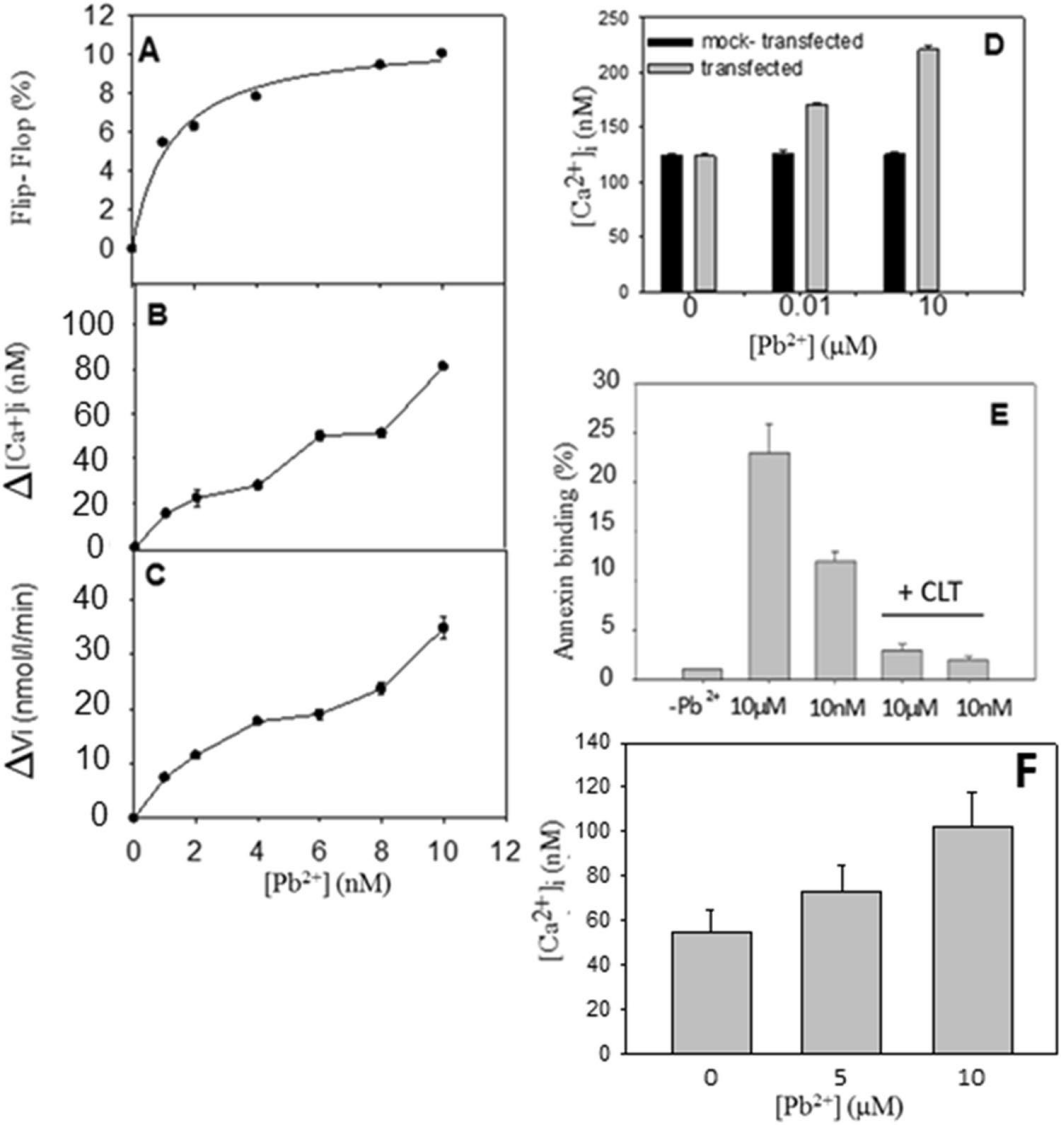
Effect of Pb^2+^ ions on cells. (**A**) Outward movement of fluorescent lipid analogue C6-NBD-PS in human RBC as a function of Pb^2+^ concentration. (**B**) Pb^2+^ - induced increase in intracellular Ca^2+^ concentration in human RBC and (**C**) Pb^2+^ -induced Ca^2+^ entrance rate in human RBC. (**D**) Pb^2+^ -induced increase in intracellular Ca^2+^ concentration of CHO cells. Intracellular free calcium was measured with Fura-2AM. Gray columns, scramblase-transfected cells; solid black columns, mock-transfected cells. (**E**) Flow cytometric analysis of CHO cell staining with annexin V-FITC, the inhibition of Pb^2+^ -induced phosphatidylserine exposure by channel inhibitor clotrimazole (CLT) can be quantified. (**F**) Pb^2+^ -induced increase in intracellular Ca^2+^ concentration of RBC cells for higher Pb^2+^ concentrations than in B. Intracellular free calcium was measured with Fura-2AM. Cells were incubated with Pb^2+^ for 1 h prior to measurements. Intracellular free calcium was measured with Fura-2AM. Mean values of three experiments ± S.D.

To measure the outward movement of lipids, RBC were first incubated with fluorescent lipid probe C6-NBD-PS (1-oleoyl-2-[6-[(7-nitro-2-1,3-benzoxadiazol-4-yl)amino]hexanoyl]-sn-glycero-3-phosphoserine). To ensure that at the start of the floppase activity all the probe was located in the inner leaflet, cells were back-exchanged with BSA. Flopping of NBD lipid was then measured by continuous BSA extraction as described under Materials and Methods. Our results show that exposure to Pb^2+^, in nanomolar concentrations, significantly increased the translocation of PS, in a dose-dependent manner (Fig. 1A). To test whether cell membrane scrambling was paralleled by loss of cell membrane integrity, release of hemoglobin was determined. Only a negligible fraction of erythrocytes was lysed in the presence of the Pb^2+^ concentrations used in this experiment, i.e. after 1 h exposure of erythrocytes to 10 nM Pb^2+^ less than 2% hemoglobin had been released.

Because a calcium-dependent pathway is considered to be a major mechanism for PS exposure in erythrocytes^11^, additional experiments were performed to elucidate whether intracellular calcium could be increased in erythrocytes after exposure to Pb^2+^. To this end, Fura2-AM (acetoxymetyl) fluorescence was used to measure changes in intracellular Ca^2+^ concentration following incubation of erythrocytes in the absence or presence of Pb^2+^. Intracellular calcium increased by Pb^2+^ treatment in a dose-dependent way (Fig. 1B). Pb^2+^ also increased Ca^2+^ entrance rates (Fig. 1C). Increased intracellular calcium levels can activate scramblase, which in turn causes PS exposure^32^. These results are in good agreement with those by Kempe, *et al*.^7^ and Shin, *et al*.^33^.

**Figure 2:**
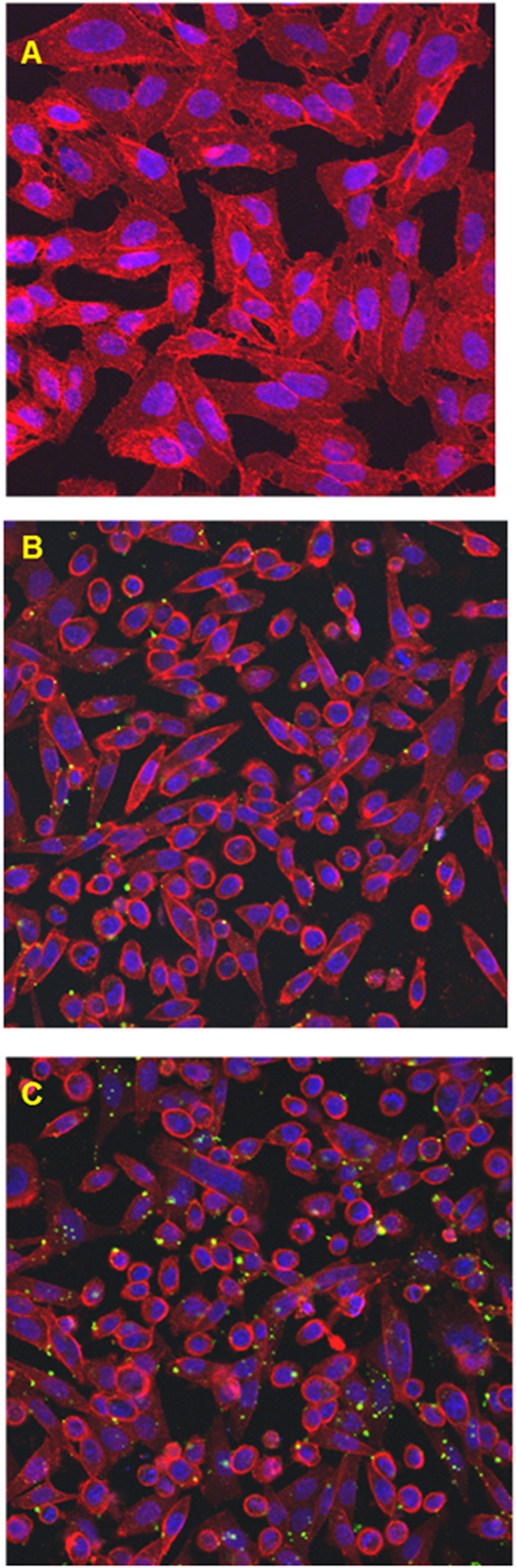
Lead-induced phosphatidylserine translocation in scramblase-transfected CHO cells. Fluorescence imaging of annexin V staining (green) in CHO cells. Annexin V staining was used to detect translocation of phosphatidylserine (PS) from the internal to the external surface of the cell membrane. Control (**A**), 10 nM Pb^2+^ (**B**), and 10 μM Pb^2+^ (**C**).

The involvement of a scramblase activity in PS translocation was demonstrated by transfection of a scramblase gene into Chinese hamster ovary (CHO) cells. CHO-K1 cells were chosen as they lack endogenous scramblase (protein scramblase 1, PLSCR1)^34^. After plasmid transfection and 24 h recovery the cells were treated with Pb^2+^. Cells were then fixed and stained. At the onset of apoptosis, PS is translocated from the cytoplasmic face of the plasma membrane to the cell surface. Annexin V has a strong affinity for PS and is therefore used as a probe for detecting early events in apoptosis. Pb^2+^ -stimulated phosphatidylserine exposure in CHO cells was observed using confocal microscopy (Fig. 2). Addition of Pb^2+^ increased annexin-binding to the cells in a dose dependent manner (Fig. 2). This result was quantified by fluorescence-activated cell sorting (FACS) analysis (Fig. 1E). As illustrated in Table 1, the results show an increase in annexin binding to transfected cells, but not to non-transfected cells. To confirm the involvement of calcium in scramblase activation, intracellular Ca^2+^ was measured as described under Materials & Methods. The results show an increase in intracellular Ca^2+^ concentration of transfected CHO cells (Fig. 1D) and in RBC (Fig. 1B), which also happens at higher Pb^2+^ concentrations in a dose-dependent way (Fig. 1F).

**Table 1:** Flow cytometric analysis of CHO cell staining with annexin V-FITC.

	Fluorescence intensity (a.u.)
CHO-pcDNA3.1 + 10 nM Pb^2+^	2.2 ± 0.20
CHO-pcDNA3.1 + 10 µM Pb^2+^	2.2 ± 0.87
CHO-Scr (Control,no Pb^2+^)	2.9 ± 0.54
CHO-Scr + 10 nM Pb^2+^	21.4 ± 1.13
CHO-Scr + 10 µM Pb^2+^	59.5 ± 1.77

Scr-transfected and mock-transfected cells were grown in the presence or absence of lead, treated with annexin V-FITC, and then analyzed by FACS. Mean values ± S.D. (n = 3).

### Morphological changes in RBC

**Figure 3:**
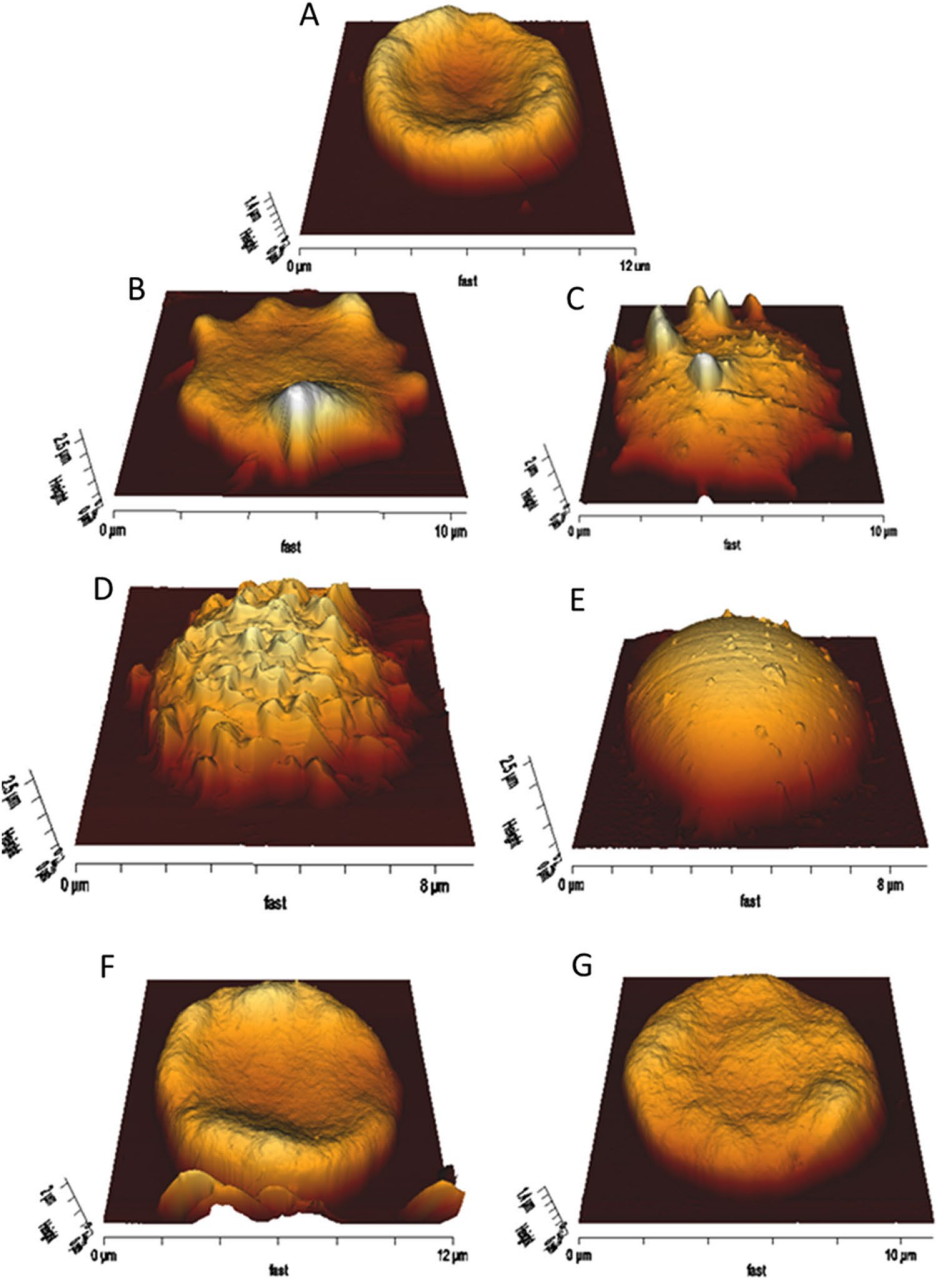
AFM monitorization of 10 µM Pb^2+^ effect on RBC. Exposure times: 0 min (**A**), 6 min (**B**), 30 min (**C**), 45 min (**D**), 60 min (**E**). Lead (II) effect on CLT-treated RBC, 20 min (**F**) and 15 h (**G**).

Atomic Force Microscopy (AFM) imaging of RBC was achieved under conditions that provided consistent information, without the risk of dragging the cells along with the cantilever tip. Figure 3 shows the time-course of 10 µM Pb^2+^ effects on RBC morphology. The characteristic biconcave shape is lost in the first minutes, and the cells become almost flat (Fig. 3B). Spikes appear and increase in number in the next ~40 min (echynocytes) (Fig. 3C,D). Then after ~1 h the cells become spherical, and the spikes are lost (spherocytes) (Fig. 3E). At the latter stage cells begin to lyse, and their number under the AFM decreases gradually, as spherocytes are prone to hemolysis^35^. The data in Table 2 show that after 1 h Pb^2+^ treatment the diameter of the RBC decreases by about 20% while the maximum thickness increases by ~87%, as expected from a discoidal-to-spheroidal shape change.

**Table 2:** Morphological changes induced by Pb^2+^ on RBC.

Pb^2+^ Time	Pre-treatment	Diameter (µm)	Maximum Thickness (µm)	Morphology
0	None	9.19 ± 0.65	1.44 ± 0.12	Doughnut
20 min	None	8.40 ± 0.46	1.10 ± 0.11	Flat/Spiked
1 h	None	7.15 ± 0.64	2.74 ± 0.28	Spherical
0	Clotrimazole	9.32 ± 0.46	1.44 ± 0.09	Doughnut
20 min	Clotrimazole	9.35 ± 0.45	1.41 ± 0.07	Doughnut
15 h	Clotrimazole	8.67 ± 0.21	1.21 ± 0.12	Doughnut/Flat
0	βMCD	8.74 ± 0.51	2.12 ± 0.16	Doughnut
40 min	βMCD	6.94 ± 0.44	3.05 ± 0.45	Spherical

Overview of the AFM data. Mean values ± S.D. (n = 50–200 cells, through 3 different experiments).

Clotrimazole (CLT) is an inhibitor of Ca^2+^ -sensitive K^+^ -channels that has been proposed as an antiapoptotic agent^36,37^. Clotrimazole, which inhibits lead-induced phosphatidylserine flip-flop (Fig. 1E) also hinders the Pb^2+^ -promoted changes in RBC morphology. CLT-treated cells retain their biconcave form even after overnight treatment with 10 µM Pb^2+^ (Fig. 3F,G). Cell diameter and maximum thickness are constant for CLT-treated RBC after 20 min 10 µM Pb^2+^ incubation (Table 2), without any statistically significant difference in diameter (p = 0.26) or thickness at the rim (‘maximum thickness’) (p = 0.86). The fact that clotrimazole inhibited RBC shape change suggests that the latter is dependent on K^+^ exit.

**Figure 4:**
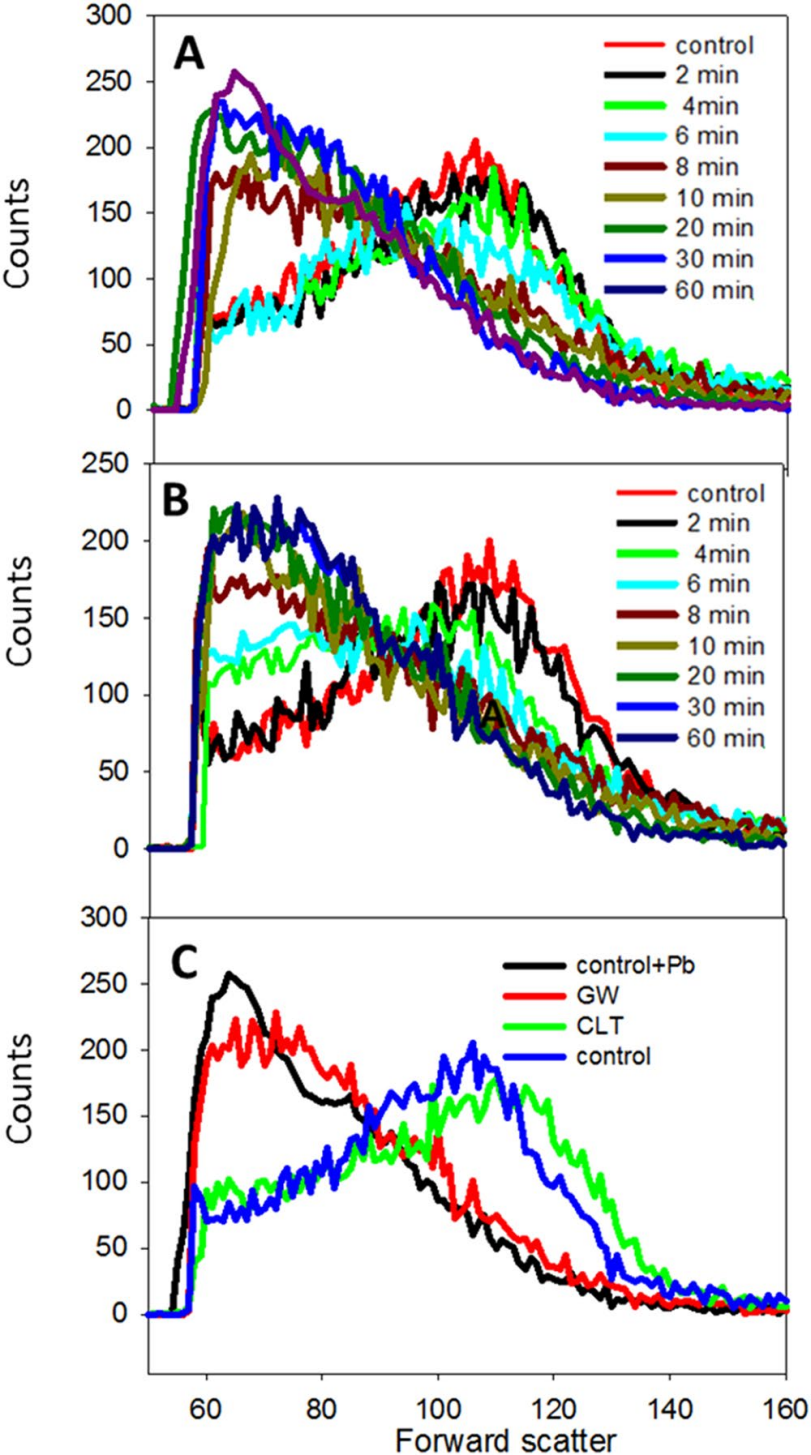
Monitorization of Pb(II) effect on RBC by FACS and the effect of inhibitors. RBC + Pb^2+^ (**A**), GW4869-treated RBC + Pb^2+^ (**B**) and comparison of control RBC (blue), +Pb^2+^ (black), GW4869-treated + Pb^2+^ (red) and CLT-treated + Pb^2+^ (green) after 60 min Pb^2+^ incubation (**C**).

**Figure 5:**
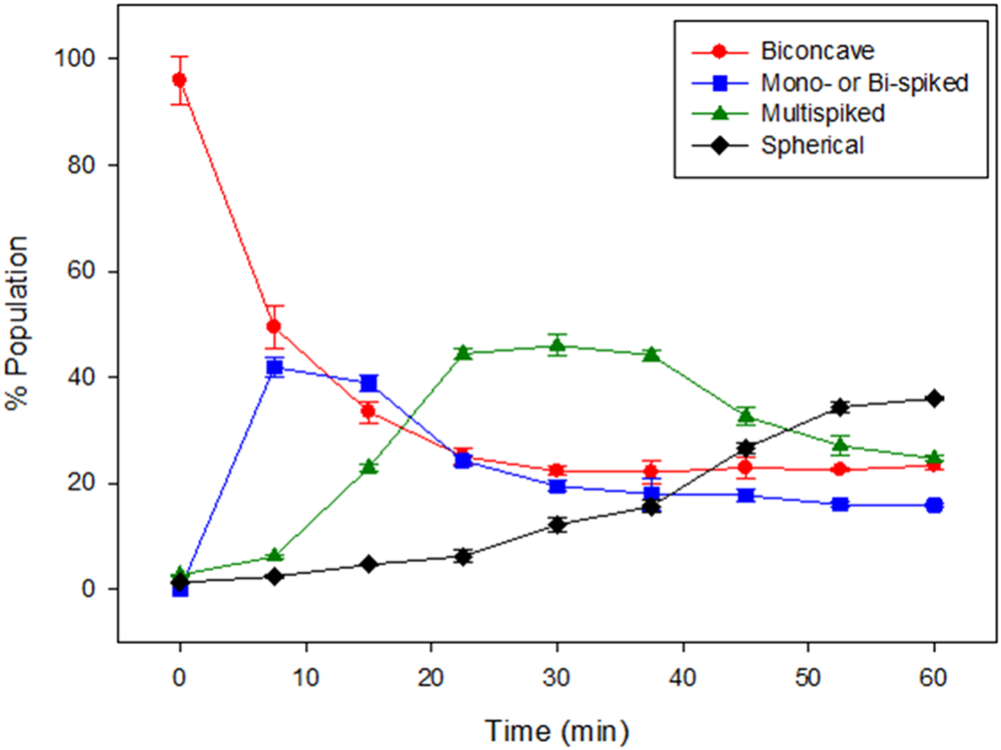
Quantitation of morphologies of RBC during 1 h Pb^2+^ 10 µM incubation process. (n = 300–450 cells).

AFM morphological changes could be confirmed for a large population of cells using flow cytometric measurements of RBC shape^38^. The results in Fig. 4 indicate for control RBC the bimodal distribution of scattered light typical of the discoidal shape. Treatment with 10 µM Pb^2+^ gave rise gradually to a more uniform scattering distribution (Fig. 4A,C), associated to sphericity^38^. In the presence of CLT the discoidal-spherical transition was not detected (Fig. 4C). The different morphologies detected during the Pb^2+^ incubation process have been quantified and presented in Fig. 5.

### Ceramide formation and cell lysis

**Figure 6:**
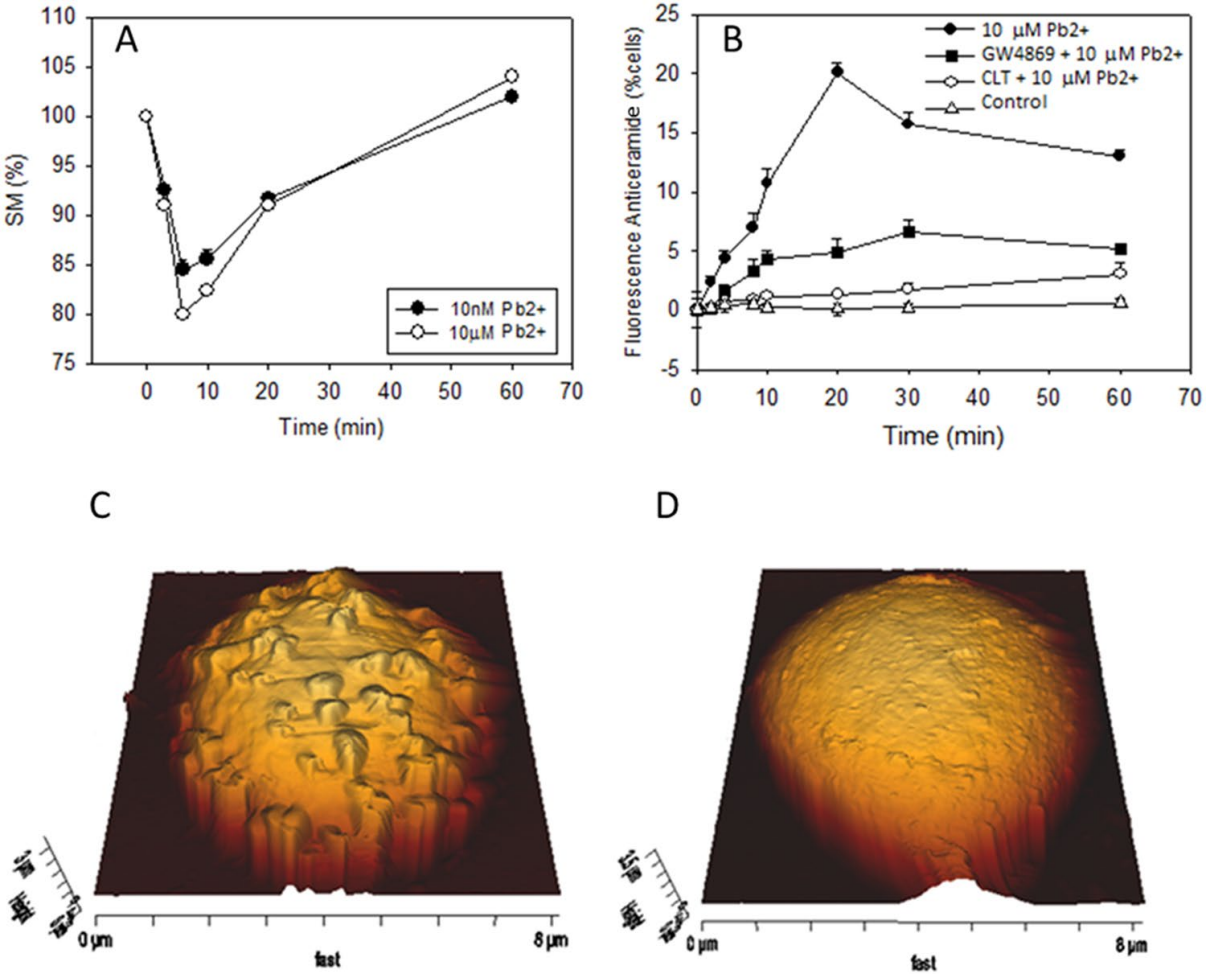
Quantitation of sphingomyelin (**A**) and ceramide (**B**) over time during Pb(II) incubation. Average of three measurements ± S.E.M. (In some cases the error bars are smaller than the symbol). Representative AFM images of GW4869-treated RBC after 15 h Pb(II) exposure in the absence of lysis (**C**,**D**). For experiments in A, SM was quantified using TLC^39^. For the experiments in B, FACS was used.

The mechanism of lead-dependent cell lysis was explored next. In a previous study we had noted the role of ceramide-enriched domains in RBC lysis^24^. We had also observed that mechanical stress elicited a neutral sphingomyelinase activity in erythrocytes^39^. We reasoned that Pb^2+^ -induced morphological changes could perhaps induce a sphingomyelinase (SMase) activity in the cell membrane. In fact, a Pb^2+^ - induced SM-ase activity was found (Fig. 6A) very similar to the one described by López, *et al*.^39^ under mechanical stress conditions. As in the previous case, SMase activity was maximum in the first 6–8 min after the stress onset. SMase activity was confirmed by quantitative mass spectrometry of RBC lipids, measuring the amounts of SM and Cer before and after 20 min treatment with Pb(II). In that period of time SM in RBC membranes slightly decreased from 4.84 ± 0.29 mol% to 4.47 ± 0.25 mol%, while ceramide significantly increased from 0.83 ± 0.06 mol% to 1.02 ± 0.07 mol% (p < 0.01) (average ± S.E.M., n = 9).

It has been observed in many systems, including RBC lipids^24,29^, that Cer can give rise to rigid domains in the RBC membranes. In order to confirm that this was the case with our samples, RBC were incubated overnight with Pb(II) and the corresponding lipid extracts dispersed in buffer and analysed by differential scanning calorimetry. Endotherms centred at ≈20 and 65 °C, are seen in lipid extracts from Pb(II)-treated, but not from control RBC (Supplementary Fig. 1). This kind of endotherms has been found in other instances of Cer formation in RBC^24^. Overnight treatment was carried out in order to facilitate the calorimetric observation of Cer. Note that the amount of Cer in overnight-treated cells will be higher than that present after the treatments described in Fig. 6.

The time-course of ceramide formation in the RBC membranes was estimated by labelling the cells with an anti-ceramide antibody and sorting the cells by FACS (Fig. 6B). Control and clotrimazole-treated cells gave no ceramide signal, while those treated with 10 µM Pb^2+^ indicated a maximum of ceramide contents at 20 min. Cells treated with the neutral SMase inhibitor GW4869 showed much decreased ceramide contents. With respect to cell shapes, flow cytometry confirms that, in the presence and absence of the SMase inhibitor, Pb^2+^ has identical effects on RBC shapes at least in the first 30 min (Fig. 4A,B). However, AFM images of Pb^2+^ - and GW4869-treated RBC overnight are equivalent to those found 45–60 min after Pb^2+^ addition in the absence of GW4869 (Fig. 3D,E and Fig. 6C,D) which reveals that in the presence of the inhibitor no cell lysis occurs. This suggests that SMase activity and ceramide formation are directly related to cell lysis. The involvement of SMase in the process was further tested using glutathione (GSH, 5 mM) as the SMase inhibitor^40^. Cell lysis was quantified for the three inhibitors (CLT, GSH and GW4869), and all three caused a reduction in cell lysis, their inhibitory efficiency decreasing in the order GSH > CLT > GW4869 (Table 3).

**Table 3:** Inhibition of Pb(II)-induced hemolyisis.

Inhibitor	Inhibition Efficiency (%)
None	0
GW4869 (15 µM)	59.9 ± 1.1
GSH (5 mM)	92.0 ± 8.7
CLT (10 µM)	76.3 ± 4.5

Average values ± S.E.M. (n = 3). Data obtained as detailed under Methods.

### Partial cholesterol removal accelerates morphological change

**Figure 7:**
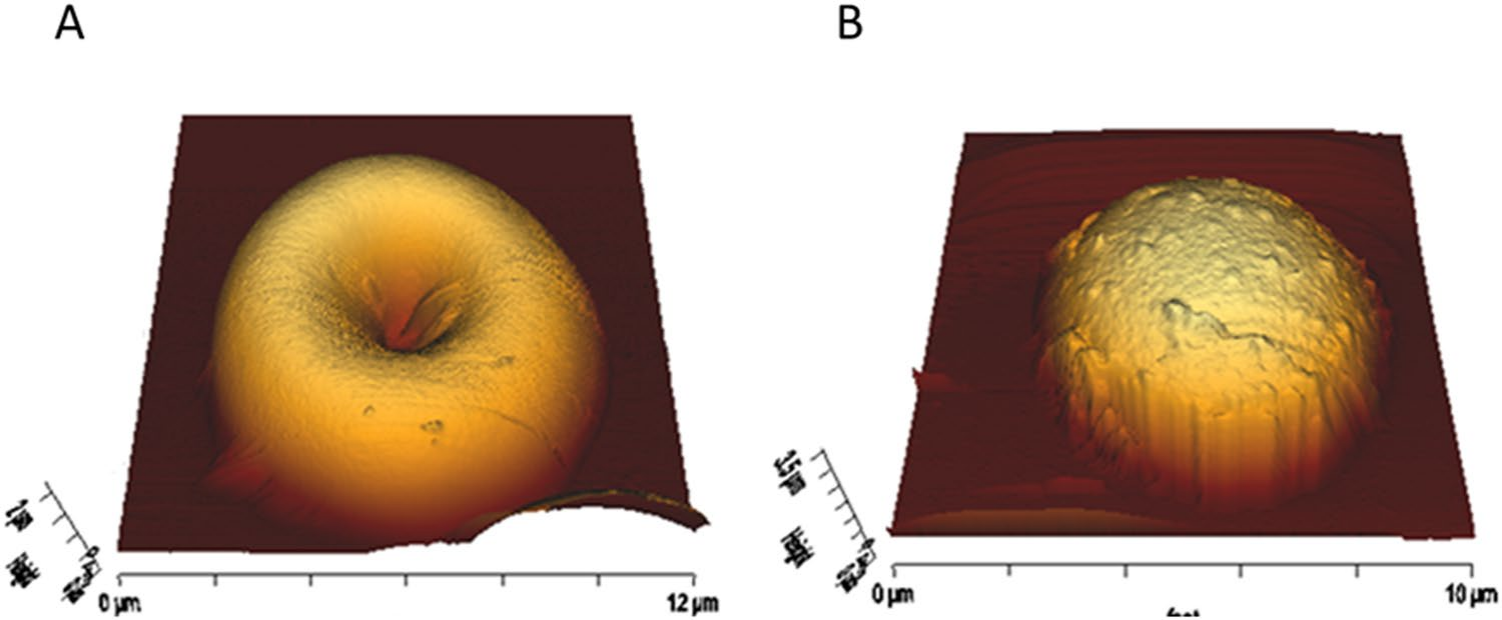
Pb^2+^ effect on βMCD-treated RBC. RBCs were incubated with 0.25 mM βMCD for 30 minutes at 37 °C for partial cholesterol depletion. AFM imaging of βMCD-treated RBCs reveals that they preserve their typical doughnut morphology (**A**). When Pb^**2+**^ is applied to them at 37 °C, spherocytes can be detected after 40 minutes (**B**), while without βMCD they took longer (>1 hour, Fig. 3E).

RBC membranes contain a high amount of cholesterol (~45 mol% lipid, in accordance with Owen, *et al*.^25^). Moreover, data from this and other laboratories suggest that a direct cholesterol-ceramide interaction may be taking place at high concentrations of both lipids^27,28^. Thus an experiment was designed to vary substantially the amount of cholesterol in RBC membranes but without causing major morphological effects *per se*. Treating the cells with 0.25 mM β-methylcyclodextrin (βMCD) under the conditions described in the Methods section RBC cholesterol (Chol) contents decreases by 31.5 ± 3.5% (n = 3) in agreement with data in the literature^41^. βMCD treatment preserves the typical doughnut morphology (Fig. 7A), except that the thickness at the rim (‘maximum thickness’) is higher in the βMCD-treated cells (2.1 vs. 1.4 µm, Table 2). The thickness and diameter data suggest that the mode of adhesion of the βMCD-treated cells is different so that they become less flattened in the process. When 10 µM Pb^2+^ is added the time-course of events is somehow faster, so that spherocytes are predominant after 40 min (Fig. 7B), as compared with 1 h for intact RBC. Pb^2+^ causes a similar decrease in diameter (21%) as in the native RBC, but the average increase in thickness is only 44%, from 2.1 to 3.1 µm (Table 2). The shorter times required in βMCD-treated cells after Pb^2+^ addition for spherocyte formation may be caused by a higher free Cer concentration due to lower cholesterol levels. A possible explanation for this result is that, perhaps, under these conditions ceramide-rich domains would be more easily formed. Another possibility is that hypothetical complex gel-like phases enriched in both ceramide and cholesterol become more Cer-enriched, as their properties are modulated by Cer: Chol ratio. This would be in agreement with the results observed in RBC lipid extract experiments^29^, as RBC lipid extracts in the presence of Cer show Cer-enriched domains which, after Chol depletion, stiffen significantly. Chol – Cer interactions have been further described in a recent review^42^.

### Fluorescence microscopy studies

**Figure 8:**
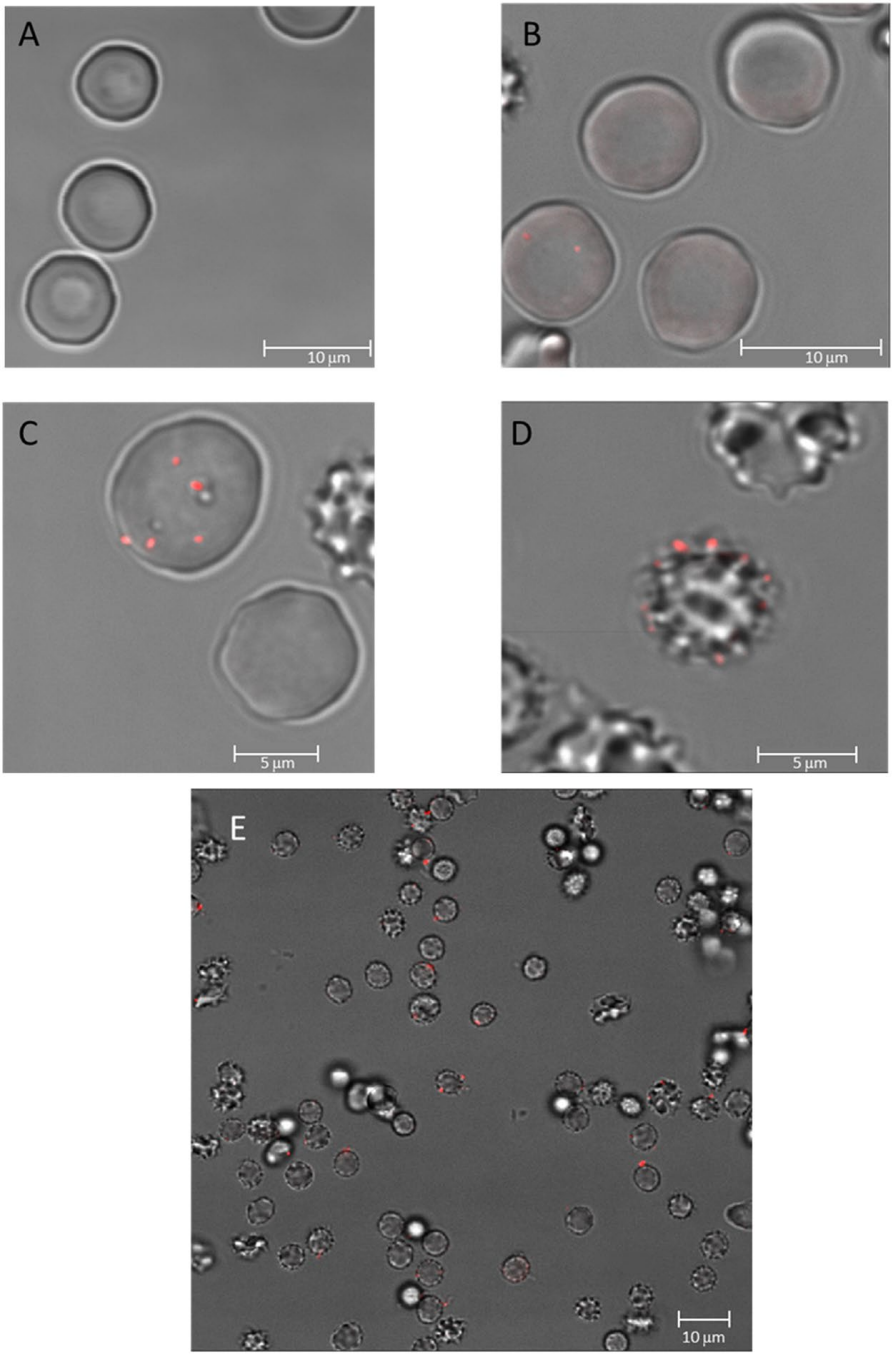
Confocal microscopy monitorization of ceramide generation over time during Pb^2+^ incubation. Exposure times: 0 min (**A**), 8 min (**B**), 10 min (**C**), 20 min (**D**,**E**). Images are a combination of transmitted light and fluorescence confocal microscopy, where red dots indicate ceramide-enriched zones detected by the antiCer- antibody (Alexa Fluor 633 as secondary antibody).

Confocal fluorescence microscopy studies were carried out with the aim of detecting and localizing ceramide and externalized PS. The former was labeled with an antiCer specific antibody (ALX-804–196 as primary, Alexa Fluor 633 as secondary), while the latter was identified through the ability of PS to bind Annexin V. Ceramide (pseudocolour red) appears in Fig. 8 as localized dots, whose number increases for 20 min after Pb^2+^ addition, in agreement with the FACS data (Fig. 4B). Ceramide is known to give rise to rigid microdomains as soon as it is formed by hydrolysis of SMase^24,43,44^. ALX-804–196 specificity against ceramide has been questioned^45^. However in our hands the antibody binds Cer with much higher affinity than phosphatidylcholine (Supplementary Fig. 2).

**Figure 9:**
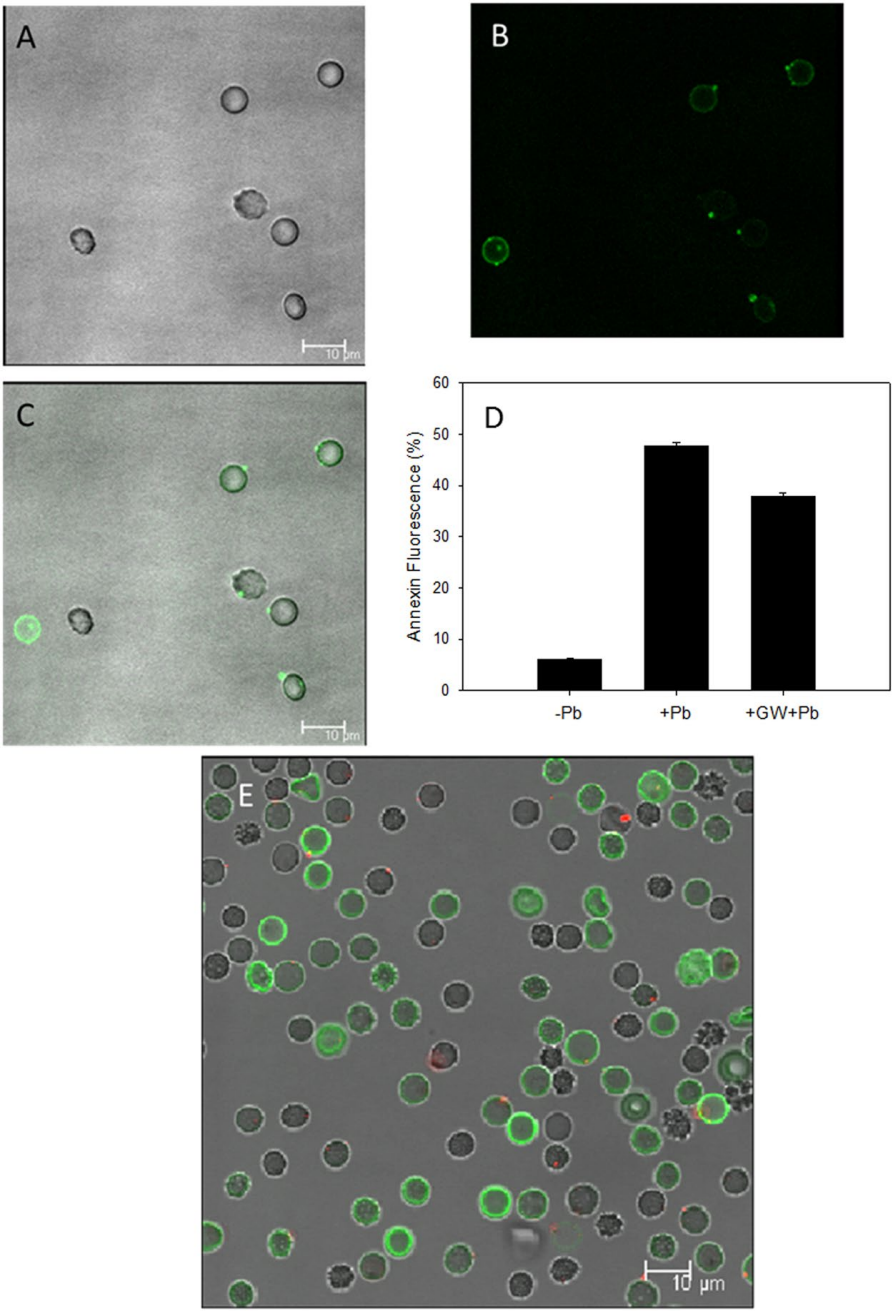
Confocal microscopy visualization of PS exposure after 20 min Pb^2+^ incubation. Annexin V was used to detect PS translocation and detected by fluorescence (green). Transmitted light (**A**), fluorescence (**B**) and mix of both images (**C**). Percentage of cells that expose PS using FACS (three different experiments, 10000 cells per experiment) (**D**). RBC treated with both annexin V and antiCer (Alexa Fluor 633 as secondary antibody) (**E**). Images in C and E are a combination of transmitted light and fluorescence confocal microscopy, where red dots indicate ceramide-enriched zones detected by the antiCer antibody as in Fig. 8.

Annexin V (pseudocolour green) indicates the presence of PS in the RBC plasma membrane outer monolayer. Figure 9A–C show the expected external location of annexin V in Pb^2+^ -treated RBC after 20 min. This is particularly clear in Fig. 9B, which shows as well the presence of single “poles” enriched in annexin V in the cells. The meaning of these hot spots of scramblase activity is not clear at present. Figure 9D includes a quantitation of externalized PS using FACS. An overlay of the ceramide and annexin V signals 20 min after Pb^2+^ addition is shown in Fig. 9E. Not all cells displaying extensive annexin V binding contain also ceramide dots. This can be due to the lack of three-dimensional imaging in these samples.

## Discussion

Phospholipid scramblases (PLSCRs) are a group of single-pass plasma membrane proteins that would carry out Ca^2+^ -dependent, non-specific and bidirectional movement (scrambling) of phospholipids^46,47^. This randomization of plasma membrane phospholipids leads to the surface exposure of PS that is an essential requirement for phagocytosis, blood coagulation and activation of anti-inflammatory responses in eukaryotic cells^48–51^. In particular, PS translocation from the inner to the outer leaflet of the plasma membrane is critical for recognition and clearance of apoptotic cells by macrophages^52,53^.

The role of PLSCR in apoptosis remains unclear given the presence of additional enzymes associated with plasma membrane phospholipid translocation such as aminophospholipid translocase^32,54–56^. Moreover, PLSCR1 appears to play important roles at the nuclear level, apparently unrelated to membrane lipid motions^57,58^. PLSCR family members contain a conserved calcium-binding motif, and Zhou, *et al*.^59^ found that mutation of residues in this region of PLSCR1 completely removed enzymatic activity. PLSCR1 is phosphorylated at Thr-161 by PKC-δ, which translocates to the plasma membrane during apoptosis^34^.

Our results show that Pb^2+^ increases intracellular Ca^2+^ (Fig. 1B,C,D). This is in agreement with the observations by Kempe, *et al*.^7^. These authors attributed the increased cytosolic Ca^2+^ concentration to either a Pb^2+^ -induced cell shrinkage, activation of an undetected Ca^2+^ -channel, or inhibition of Ca^2+^ -ATPase. The latter enzyme is known to be inhibited by Pb^2+ ^^60–62^. Earlier work shows that the cytoplasmic Ca^2+^ concentration and induction of apoptosis are directly related to each other^48–50^. A change in conformation of PLSCR1 upon Ca^2+^ binding has been proposed by Stout, *et al*.^63^. Other results^64^ show that the ionic interaction of Ca^2+^ with PLSCR1 peptides lead to a change in conformation of the protein around the Ca^2+^ binding motifs. However, free cytosolic Ca^2+^ may also act as a second messenger and activate many cytosolic proteins involved in apoptotic pathways^64^. Lang’s group^7^ has demonstrated that [Pb^2+^] > 0.1 µM also activates erythrocyte K^+^ channels, leading to erythrocyte shrinkage, and to activation of the erythrocyte scramblase, causing PS exposure. Pb^2+^ ions have been demonstrated to directly activate Ca^2+^ -sensitive Gardos K^+^ -channels (BK channels) in human erythrocytes^65^ and similar Ca^2+^ -sensitive K^+^ -channels in other cell types^66,67^. However, the fact that we have found that Pb^2+^ induced Ca^2+^ entry and PS exposure in CHO cells only when the latter had been transfected with PLSCR1 gene (Figs 1 and 2, and Table 1) is a direct demonstration that scramblase is involved in Pb^2+^.-induced PS flopping. In addition, alternative Ca^2+^ -independent mechanisms of PLSCR1 activation have been suggested under certain conditions^68^.

Clotrimazole is an inhibitor of BK channels. It has been found to inhibit Pb^2+^ -induced PS flip-flop motion and annexin V binding (Fig. 1E). The inhibition is largely due to a channel blockade^69^. BK channels open up when the intracellular Ca^2+^ concentration increases, and allow the efflux of intracellular K^+^ to the extracellular medium^70^. Clotrimazole also inhibits the Pb^2+^ -induced morphological changes in the RBC, which suggests a role for the perturbation of ionic concentration in the shape changes. Dreher, *et al*.^71^ reported that modest increases in intracellular Ca^2+^ concentrations caused the appearance of pathologic changes in erythrocyte shape and deformability. However these changes were prevented when the cells were suspended in high-K^+^ buffers, suggesting a role for K^+^ in the shape changes. Glaser, *et al*.^72^ and Bifano, *et al*.^73^ have also related shape changes to membrane potential in RBC, thus to K^+^ concentrations and fluxes. Bifano, *et al*.^73^ observed that increased intracellular Ca^2+^ enhanced K^+^ conductance and cell membrane hyperpolarization. Under these circumstances these authors observed echinocytosis, just as in our case (Fig. 3B–D). Clotrimazole blocks the K^+^ channels and no echinocytosis is observed (Fig. 3F,G).

**Figure 10:**
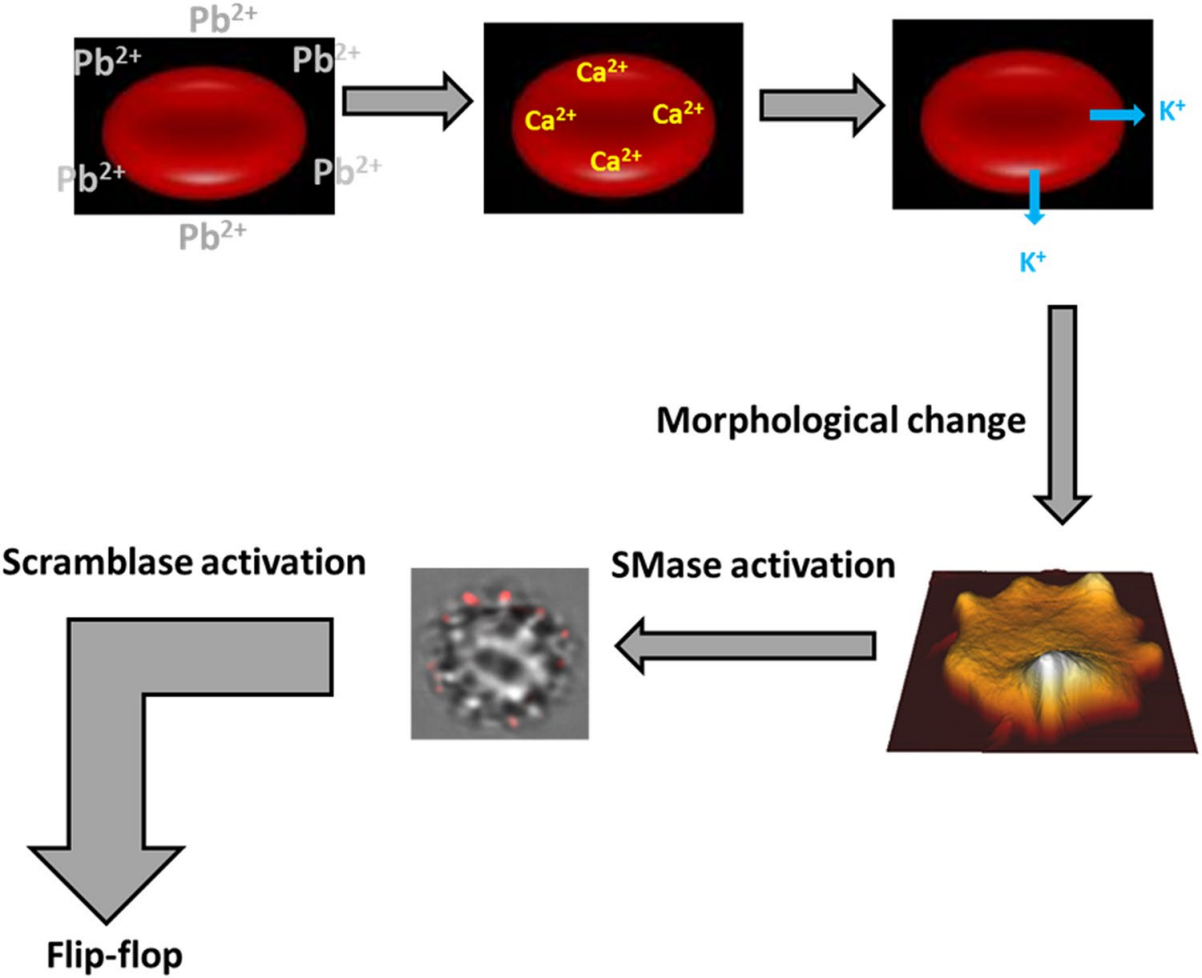
Pb^2+^ -induced changes on RBC. General outline of the process.

Echinocytosis is very likely to elicit the SMase activity described by López, *et al*.^39^, as any other membrane bending process, thus a cause-effect relationship is observed between Pb^2+^, increase in intracellular Ca^2+ ^^7^, K^+^ outflow^71^, echinocytosis^73^, and SMase activation^39^. SMase activity is found in our cells under conditions causing echinocytosis (Fig. 6A). The generation of ceramide could explain the reported increase in the order of the membranes by the effect of Pb^2+ ^^16^, which could act through membrane platforms (Fig. 8). SMase inhibition by GW4869 does not inhibit echinocytosis (Fig. 5C) but CLT does inhibit spike formation, and CLT inhibits both echinocytosis and SMase activity (Figs 3F and 6B). This supports the flow of information summarized in Fig. 10. Cholesterol depletion accelerates the morphological change and this could be related to ceramide-enriched platforms, as less cholesterol promotes their generation and could stiffen the domains. However, recent reports indicate that scramblase 1 could also interact with cholesterol^74^, and the possibility of cholesterol depletion enhancing scramblase activity cannot be discarded.

The last step in this series of events would be the PS exposure to the outer membrane as a result of flip-flop phospholipid motion. As far as we know, flip-flop can occur either as a result of SMase-dependent ceramide formation^17,18^, or through the action of a scramblase, or both. Human scramblase 1 has been shown to catalyze phospholipid flip-flop in the absence of ceramide^15,47,75,76^. In our hands, SMase inhibition by GW4869 partially inhibits PS externalization (Fig. 9D), while inhibition of Ca^2+^ entry, essential for scramblase activation, by clotrimazole abolishes all downstream changes. These data suggest that scramblase and ceramide act jointly towards the membrane lipid scramblase effect, and subsequent externalization of PS.

Our results also support the idea, proposed by Kempe, *et al*.^7^, that scramblase activation is at the origin of the observed anemia after Pb^2+^ intoxication. Recent findings suggest that PS exposure also occurs under non-apoptotic conditions. Many pathological conditions cause surface exposure of PS, suggesting activation of PLSCRs^77^. Cytosolic Ca^2+^ is known to increase in several conditions (e.g. thalassemia, sickle cell disease, glucose-6-phosphate dehydrogenase deficiency) where scramblases need to be activated and the rate of apoptosis is increased^78,79^. In the light of the above results, lead could be an aggravating factor in those diseases.

In conclusion, in the present work we show evidence of the effects of Pb^2+^ ions on red blood cells, and we characterize the mechanism of this eryptotic process. Pb^2+^ causes Ca^2+^ entry which in turn causes K^+^ depletion from the cell. This process can be stopped at this point using clotrimazole. After K^+^ depletion, the red blood cells undergo a morphological change that initially flattens the cell and leads to the generation of spikes (echinocytosis), and finally to spherocytes. Ceramide generation is linked to the morphological change and the process can be stopped at this point using GW4869 neutral sphingomyelinase inhibitor. Finally, the cell activates a scramblase and ‘flip-flop’ lipid motion occurs, exposing PS from the inner leaflet of the cell membrane to the outer monolayer, and leading to hemolysis. This work is relevant in the context of cell death, heavy metal toxicity and sphingolipid signaling.

## Materials and Methods

### Materials

Lead acetate, Fura-2AM, EMEM (Essential Minimum Eagle Medium), BSA (bovine serum albumin), Clotrimazole and GW4869 were obtained from Sigma (St Louis, MO, USA). Annexin V-fluorescein isothiocyanate conjugate (FITC) Kit was purchased from Molecular Probes (Eugene, OR, USA). Alexa Fluor 633 goat anti-mouse IgM, Lipofectamine 2000 and pc DNA 3.1 were from Invitrogen (Carlsbad, CA, USA). 1-oleoyl-2-[6-[(7-nitro-2-1,3-benzoxadiazol-4-yl)amino]hexanoyl]-sn-glycero-3-phosphoserine (C6-NBD-PS) was obtained from Avanti Polar Lipids Alabaster, AL. ALX-804-196 Ceramide, mAb (MID 15B4) (“antiCer” antibody) was purchased from Enzo Life Sciences, Inc. (Farmingdale, NY, USA). All other reagents and solvents for assay were of analytical reagent grade and were purchased from Sigma-Aldrich.

### Isolation of RBC

Whole blood was obtained from informed healthy volunteers by venipuncture. RBC were collected by centrifugation (1700 g, 10 min) and resuspended in RBC buffer (32 mM HEPES, 125 mM NaCl, 1 mM MgSO_4_, 1 mM CaCl_2_, 5 mM KCl, 5 mM glucose, pH 7.2). The process was repeated two more times to increase efficiency. The haematocrit level was adjusted to 1% (A_412_ ≈ 0.6 for a 25 µl -on- 1 ml distilled water dilution).

### Labeling of RBC with C6-NBD phospholipids

Washed human RBC were resuspended in RBC buffer to a cell concentration of 5.10^8^ cells/mL and loaded with 2 µM C6-NBD-PS. Translocation of the probe proceeded for 1 h at 37 °C, resulting in 75–95% internalization. To measure outward movement, residual C6-NBD-lipid remaining in the cells outer monolayer was removed by washing for 5 min with ice-cold 0.5% BSA prior to the experiment.

### Translocation of RBC membrane lipids from the inner to the outer leaflet

Outward movement of C6-NBD-PS was measured using the BSA back-exchange procedure as described by Connor, *et al*.^80^. Labelled RBC were incubated with different Pb^2+^ concentrations at 37 °C. Briefly, 200 µL aliquots from the cell suspension were removed at the indicated time intervals and placed on ice for 5 min in the presence or absence of 1% BSA. Pellets obtained after 3 min centrifugation at 12000 *g* were solubilized in 2 mL 1% (w/v) Triton X-100, and the amount of externalised probe was determined by comparing the fluorescence intensity associated with the cells before and after back-exchange. The amount of probe extracted into BSA was related to the control sample incubated without Pb^2+^, that was established as 100%. The fluorescence assay was measured at 37 °C, using an SLM Aminco 8100 spectrofluorometer, equipped with a circulating water bath. The excitation and emission wavelengths were 480 and 525 nm respectively.

### Hemolysis assay

The hemolysis assay was performed in 1-ml test tubes by mixing the erythrocyte suspension (*A*_412_ ≈ 0.6) with the required lead concentrations. Inhibitors were applied previously in the corresponding samples and preincubated for 1 hour. The mixtures were incubated at 37 °C for 1 h in the presence of Pb(II) with gentle shaking. After centrifugation at 1700 *g* for 5 min, hemolytic activity was measured as the increase in *A*_412_ (i.e., increase in hemoglobin content) of the supernatant (Equations 1 and 2). Inhibition efficiency was calculated following Equation 3.

1 $$ \% {lysis}=\frac{{{A}}_{412,{supernatant}}\,}{{{A}}_{412,{resuspended}}}\cdot 100$$

2 $${\rm{\Delta }} \% lysis= \% lysi{s}_{sample}- \% lysi{s}_{control}$$

3 $$Inhibition\,Efficiency\,( \% )=\,\frac{{\rm{\Delta }} \% lysi{s}_{Pb}-{\rm{\Delta }} \% lysi{s}_{Pb+inh}}{{\rm{\Delta }} \% lysi{s}_{Pb}}\,\cdot 100$$

Δ%lysis_Pb_ stands for the increase in cell lysis for the Pb-containing sample without inhibitor, and Δ%lysis_Pb+inh_ stands for the increase in cell lysis for the Pb + inhibitor sample. Complete inhibition of the process (Δ%lysis_Pb+inh_ = 0) would render a 100% inhibition efficiency, while the opposite case, a lack of effect of the inhibitor (Δ%lysis_Pb+inh_ = Δ%lysis_Pb_), would render a 0% inhibition efficiency.

### Measurement of cell [Ca^2+^]_i_

Cells were loaded with 4 µM Fura2-AM at 37 °C for 30 min. Excess Fura2-AM was removed by rinsing twice with buffer. Both the cells undergoing Pb^2+^ treatment and the control group were resuspended in buffer. Fluorescence intensity was monitored at an emission wavelength of 510 nm, by using a pair of excitation wavelengths at 340 nm and 380 nm, and subsequently expressed as the ratio of light excited at 340 nm to that at 380 (F_R_). Cells were permeabilized with 0.03% Triton X-100 and F_max_ was obtained. EGTA (3 mM final concentration) was then added and F_min_ was obtained. Sf_2_/Sb_2_ is the ratio between the excitation efficiencies of free probe and Ca^2+^ -bound probe at 380 nm. [Ca^2+^]_i_ was calculated according to the equation of Grynkiewicz, *et al*.^81^ (Equation 4).

4 $${[{{\rm{Ca}}}^{2+}]}_{{\rm{i}}}=\frac{\mathrm{Kd}\,({{\rm{F}}}_{{\rm{R}}}-{{\rm{F}}}_{{\rm{\min }}})}{({{\rm{F}}}_{{\rm{\max }}}-{{\rm{F}}}_{{\rm{R}}})}\frac{{{\rm{Sf}}}_{2}}{{\mathrm{Sb}}_{2}}$$

Where Kd is 371 ± 71 nM.

### Cell culture

Chinese hamster ovary CHO-K1 cells were obtained from the American Type Culture Collection. These cells were maintained in EMEM containing 10% foetal bovine serum and 5000 U/ml penicillin and streptomycin under a humidified atmosphere of 5% CO_2_ in air.

### Transfection of CHO cells

CHO-K1 cells, grown to 70–80% confluence, were transfected with pcDNA-PLSCR1 or pcDNA-3/1 (the latter lacking the scramblase gene) using LIPOFECTAMINE^TM^ 2000 reagent according to the manufacturer’s instructions. Transfected cells were grown for 24 h before analysis. 5 h after transfection cells were switched to growth medium containing 350 µg/ml Geneticin antibiotic (G418) and the selection medium was refreshed every 24 h.

### Mass spectrometric analysis of RBC sphingolipids

Lipid extraction was performed using a modified MTBE protocol (see Guri, *et al*.^82^). Briefly RBC were transferred into a 2 mL Eppendorf tube. Then, 360 μl methanol was added and vortexed. A mixture of lipid standards (see Table 4) was added and the cells were vortexed for 10 min at 4 °C using a Cell Disruptor Genie (Scientific Industries, Inc., Bohemia, NY). MTBE (1.2 mL) was then added and the samples were incubated for 1 h at room temperature with shaking (750 rpm). Phase separation was induced by adding 200 μl H_2_O. After 10 min incubation at room temperature, the sample was centrifuged at 1000 × g for 10 min. The upper (organic) phase was transferred to a 13 mm screw-cap glass tube and the lower phase was extracted with 400 μl artificial upper phase (MTBE/methanol/water (10:3:1.5, v/v/v)). The two upper phases were combined and the total lipid extract (in a 13 mm glass tube) was dried in a Centrivap at 50 °C or under a nitrogen flow. The aliquot was deacylated to eliminate phospholipids by methylamine treatment (Clarke method). 0.5 mL monomethylamine reagent (MeOH/H2O/n-butanol/Methylamine solution (4:3:1:5 v/v) was added to the dried lipid, followed by sonication (5 min). Samples were then mixed and incubated for 1 h at 53 °C and dried (as above). The monomethylamine-treated lipids were desalted by *n*-butanol extraction. 300 μl H_2_O-saturated n-butanol was added to the dried lipids. The sample was vortexed, sonicated for 5 min and 150 μl MS-grade water was added. The mixture was vortexed thoroughly and centrifuged at 3200 × g for 10 min. The upper phase was transferred to a 2 mL amber vial. The lower phase was extracted twice more with 300 μl H_2_O-saturated *n*-butanol and the upper phases were combined and dried (as above).

**Table 4:** Standards and conditions for mass spectrometric analysis of RBC sphingolipids.

Lipid Class	Standard	Polarity	Mode	m/z ion	Collision Energy (eV)
Ceramide	C17Cer	+	Product ion	264.34	25
Dihydroceramide	C17Cer	+	Product ion	266.34	25
Hexosylceramide	C8GC	+	Product ion	264.4	30
Hexosyldihydroceramide	C8GC	+	Product ion	266.4	30
Sphingomyelin	C12SM	+	Product ion	184.07	26

Sphingolipids were detected on a Triple Quadrupole TSQ Vantage mass spectrometer (Thermo Fischer Scientific, Waltham, MA). Sphingolipid aliquots were resuspended in 250 μl chloroform/methanol (1:1 v/v) (LC-MS/HPLC grade) and sonicated for 5 min. The samples were pipetted into a 96-well plate (final volume = 100 μl). The samples were diluted 1:10 in positive mode solvent (Chloroform/Methanol/Water (2:7:1 v/v) + 5 mM ammonium acetate) and infused into the mass spectrometer. Tandem mass spectrometry for the identification and quantification of sphingolipid molecular species was performed using Multiple Reaction Monitoring (MRM) with a TSQ Vantage Triple Stage Quadrupole Mass Spectrometer (Thermo Fisher Scientific) equipped with a robotic nanoflow ion source, Nanomate HD (Advion Biosciences, Ithaca, NY). The collision energy was optimized for each lipid class. The detection conditions for each lipid class are listed below (Table 4). Ceramide species were also quantified with a loss of water in the first quadrupole. Each biological replicate was read in 2 technical replicates (TR). Each TR comprised 3 measurements for each transition. Lipid concentrations were calculated relative to the relevant internal standards and then normalized to the total lipid content of each lipid extract (mol%).

### Assays for PS externalization and ceramide domain visualization

Cells grown on glass coverslips were rinsed with PBS and incubated with different concentrations of Pb^2+^. Annexin-V-FITC staining was performed according to the manufacturer’s instructions. After rinsing coverslips with binding buffer to remove unbound annexin-V, cells were fixed with 3% paraformaldehyde for 15 min and rinsed twice with PBS. As for ceramide domain visualization, cells were incubated with antiCer, centrifuged at 1700 g for 10 min to remove supernatant (cells were resuspended with RBC buffer) and reincubated with Alexa Fluor 633 followed by an additional centrifugation step. Cells were resuspended with RBC buffer, then fixed with 3% paraformaldehyde. Coverslips were mounted on glass slides. Confocal images were acquired with an Olympus Fluoview FV500 confocal microscope.

### Fluorescence-activated cell sorting analysis

Fluorescence-activated cell sorting (FACS) analysis was performed as described previously^83^. After incubation in the presence or absence of Pb^2+^ cells were stained with Annexin-V-FITC and fluorescence was measured using flow cytometric analysis (FACS Calibur; Becton-Dickinson, Franklin Lakes, NJ). For each sample, the fluorescence from 10,000 events was acquired for data analysis and the results were expressed as the mean fluorescence intensity of cells, selected in a forward- versus side-scatter window. Annexin fluorescence intensity was measured in fluorescence channel FL-1 with λ_ex_ = 488 nm and λ_em_ = 530 nm, while for ceramide detection λ_ex_ = 625 nm and λ_em_ = 660 nm was used. All measurements were performed in triplicate. These experiments were performed in the Flow Cytometry Service of the University of the Basque Country (UPV/EHU).

### RBC preparation for atomic force microscopy (AFM) imaging

Red blood cell suspension was prepared by 80-fold dilution of the 1% haematocrit-adjusted suspension in RBC buffer. This diluted suspension was set on an ethanol-washed round glass coverslip and left for 10–15 minutes for adhesion. To both facilitate AFM imaging and stop the Pb(II)-induced cell death process, 0.5% glutaraldehyde was added after the adhesion time.

### AFM imaging

Experiments were carried out at room temperature (23 °C) in a JPK Nanowizard II AFM (JPK Instruments, Berlin, Germany). Images were captured at 1 Hz with 512 × 512 pixels. For this purpose, V-shaped MLCT silicon nitride cantilevers were used (Bruker AXS, Karlsruhe, Germany) in contact mode, with enough force to reduce the overestimation of cell diameters at the edges without compromising cell integrity. Images were collected at 512 × 512-pixel resolution at a scanning rate of 1 Hz and line-fitted using the JPK Data Processing software as required. Cell diameters were measured on the images along the Y and X axis for each cell, combining both data (as RBC have almost circular shape when attached to support). Cell thickness was measured by cross-section analysis of the RBC images.

### Cholesterol partial depletion

β-methylcyclodextrin (βMCD) was prepared in RBC buffer to a 0.25 mM final concentration. RBC were incubated in this βMCD-containing buffer at 37 °C for 30 minutes to achieve partial cholesterol removal^40^. Then RBC were washed by centrifugation (1700 g, 10 min) discarding the supernatant and resuspended in βMCD-free RBC buffer. Cholesterol quantification was performed with the cholesterol oxidase/peroxidase Automated Systems Reagent (BioSystems, Barcelona, Spain) kit.

### Ethical Aspects

All methods were carried out in accordance with relevant guidelines and regulations. Informed consent was obtained from all subjects (blood donors). This study was approved by the Research Ethics Committee from the University of the Basque Country (Comité de Ética en la Investigación y la Práctica Docente de la Universidad del País Vasco/Euskal Herriko Unibertsitatea, CEID/IIEB)

## Electronic supplementary material

Supplementary Figures
