# Supplementary material for: Pb(II) Induces Scramblase Activation and Ceramide-Domain Generation in Red Blood Cells
Source: Sci Rep. 2018 May 10;8:7456. doi: 10.1038/s41598-018-25905-8 (PMC5945622; doi:10.1038/s41598-018-25905-8)
Supplement: Supplementary file 1 — Supplementary Figures [file 41598_2018_25905_MOESM1_ESM.pdf]

SUPPLEMENTARY FIGURES

Pb(II) Induces Scramblase Activation and Ceramide-Domain Generation in Red Blood Cells

Hasna Ahyayauch*[†#º], Aritz B. García-Arribas*[†§], Jesús Sot[†], Emilio J. González-Ramírez[†§], Jon V. Busto[†§], Bingen G. Monasterio[†§], Noemi Jiménez-Rojo[†§&], F. Xabier Contreras[†§], Adela Rendón-Ramírez[†§], Cesar Martin[†§], Alicia Alonso[†§] and Félix M. Goñi[†§].

[†] Instituto Biofisika (CSIC, UPV/EHU), 48080, Bilbao, Spain.

[§]Departamento de Bioquímica, University of the Basque Country (UPV/EHU), 48080, Bilbao, Spain.

[#]Institut Supérieur des Professions Infirmières et des Techniques de Santé, Rabat, Morocco.

[º]Neuroendocrinology Unit, Laboratory of Genetics, Neuroendocrinology and Biotechnology, Faculty of Sciences, Ibn Tofail University, Kenitra, Morocco.

[&]NCCR Chemical Biology, Department of Biochemistry, University of Geneva, 1211 Geneva, Switzerland

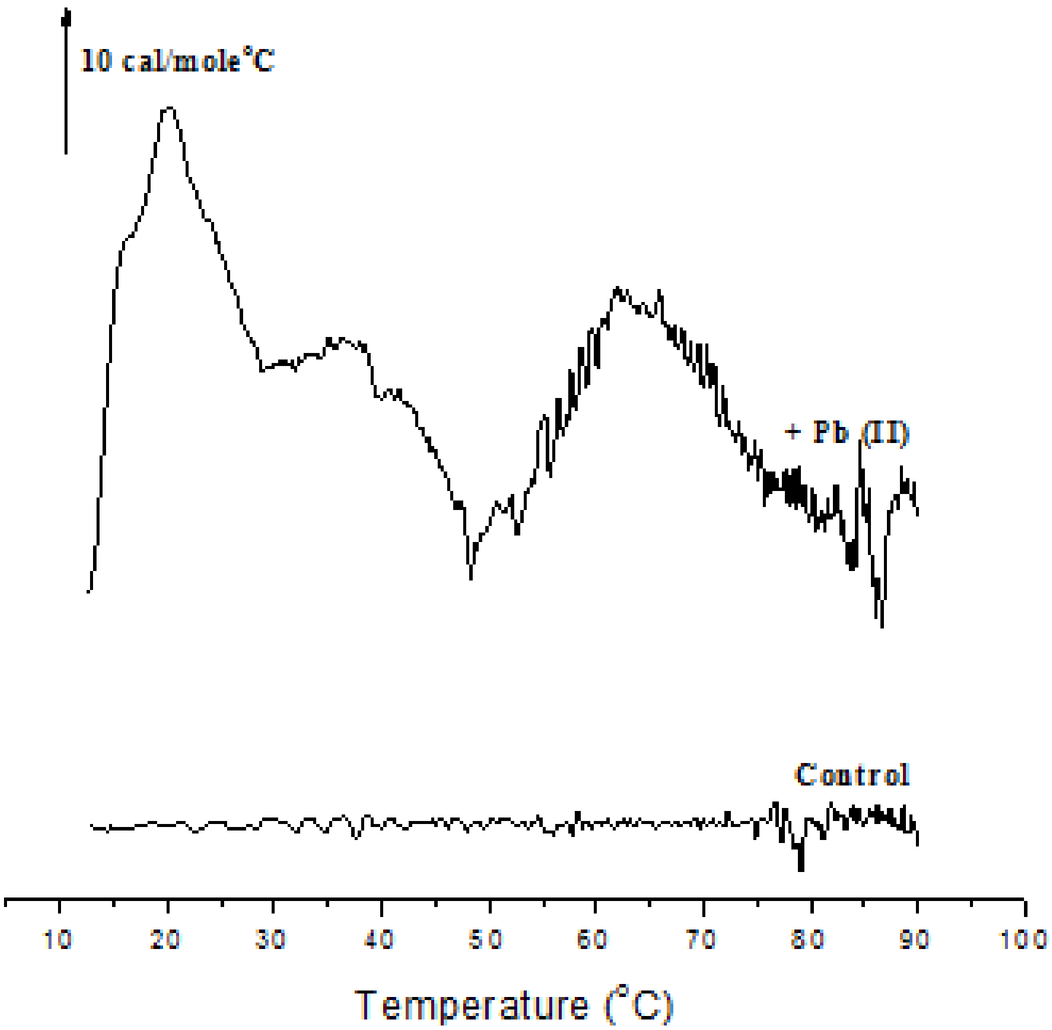

Supplementary Figure 1. Representative DSC thermograms of erythrocyte membrane lipid extracts resuspended in buffer. Bottom: control. Top: Erythrocytes treated with Pb(II) overnight. The endotherms correspond to the melting of ceramide-enriched membranes[24].

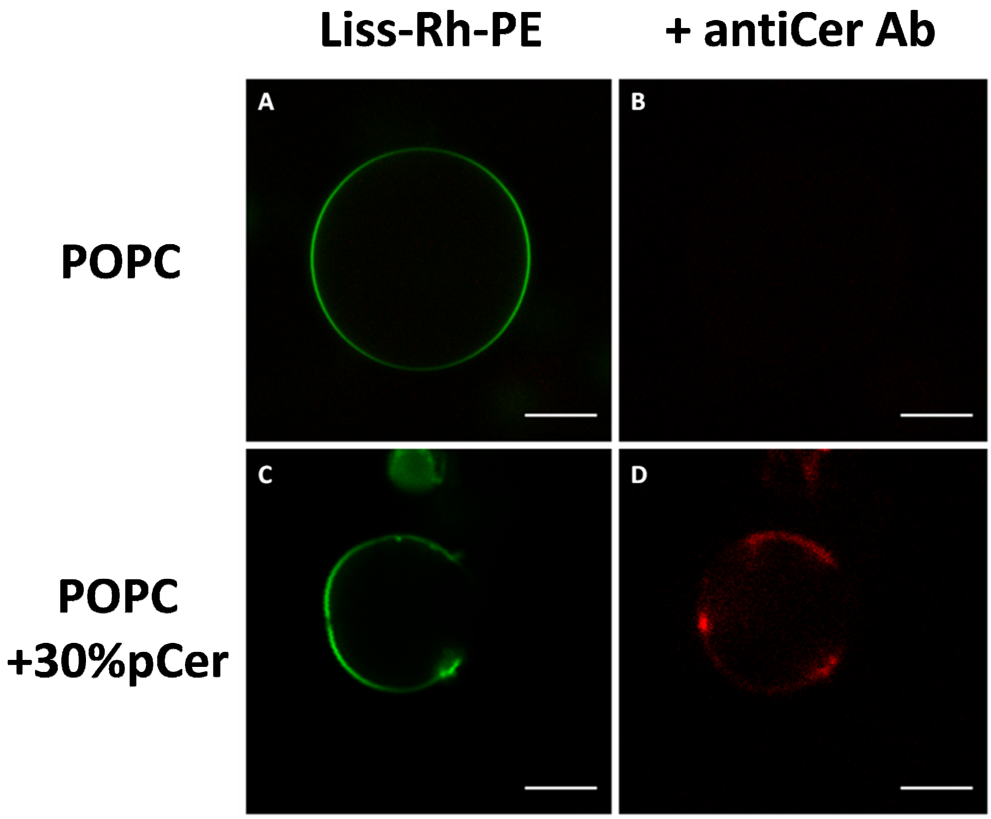

Supplementary Figure 2. Confocal microscopy images of GUVs. Direct observations of GUVs containing POPC with Liss-Rho-PE [A] and Alexa 633 [B]. Direct observations of GUVs containing POPC:pCer (80:20) with Liss-Rho-PE [C] and Alexa 633 [D]. The antibody is seen to bind ceramide-containing membranes with much higher affinity than the pure POPC bilayers.
